# A “fit for purpose” framework for medical education accreditation system design

**DOI:** 10.1186/s12909-020-02122-4

**Published:** 2020-09-28

**Authors:** Sarah Taber, Nesibe Akdemir, Lisa Gorman, Marta van Zanten, Jason R. Frank

**Affiliations:** 1 Royal College of Physicians and Surgeons of Canada, Ottawa, Canada; 2 OLVG Teaching Hospital, Amsterdam, Netherlands; 3 VU Medical Center, School of Medical Sciences, Amsterdam, Netherlands; 4 Foundation for Advancement of International Medical Education and Research, Philadelphia, PA USA

**Keywords:** Accreditation framework, Accreditation system design, Continuous quality improvement, Medical education

## Abstract

**Background:**

Accreditation is a key feature of many medical education systems, helping to ensure that programs teach and assess learners according to applicable standards, provide optimal learning environments, and produce professionals who are competent to practise in challenging and evolving health care systems. Although most medical education accreditation systems apply similar standards domains and process elements, there can be substantial variation among accreditation systems at the level of design and implementation. A discussion group at the 2013 World Summit on Outcomes-Based Accreditation examined best practices in health professional education accreditation systems and identified that the literature examining the effectiveness of different approaches to accreditation is scant. Although some frameworks for accreditation design do exist, they are often specific to one phase of the medical education continuum.

**Main text:**

This paper attempts to define a framework for the operational design of medical education accreditation that articulates design options as well as their contextual and practical implications. It assumes there is no single set of best practices in accreditation system development but, rather, an underlying set of *design decisions.* A “fit for purpose” approach aims to ensure that a system, policy, or program is designed and operationalized in a manner best suited to local needs and contexts. This approach is aligned with emerging models for education and international development that espouse decentralization.

**Conclusion:**

The framework highlights that, rather than a single best practice, variation among accreditation systems is appropriate provided that is it tailored to the needs of local contexts. Our framework is intended to provide guidance to administrators, policy-makers, and educators regarding different approaches to medical education accreditation and their applicability and appropriateness in local contexts.

## Background

Accreditation is a powerful lever of quality assurance (QA) and quality improvement (QI) in medical education. It is a key feature of many medical education systems, helping to ensure that programs teach and assess learners according to applicable standards, provide optimal learning environments, and produce professionals who are competent to practise in challenging and rapidly evolving health care systems. In addition, most medical education accreditation systems apply similar standards domains as well as similar process elements and face similar challenges and debates, regardless of jurisdiction, context, or stage of training. Accreditation and its common elements are described in further detail by Frank and colleagues [1].

Despite their similarities, there can be substantial variation among accreditation systems at the level of design and implementation. A discussion group at the 2013 World Summit on Outcomes-Based Accreditation^1^ examined best practices in health professional education accreditation systems and identified that the literature examining the effectiveness of different approaches to accreditation systems is scant. This research gap, together with inherent difficulties in evaluating the effectiveness of accreditation systems, has itself been highlighted in the literature [2, 3].

Drawing on their own experience with local accreditation systems, the members of the discussion group also noted that the substantial variation that exists in accreditation systems across jurisdictions, type of education, and stage of training often has little or no identifiable rationale. Although some frameworks for accreditation design do exist, such as the World Federation for Medical Education (WFME) Recognition Criteria for Agencies Accrediting Medical Schools [4, 5] and the quality management framework described by Akdemir and colleagues [6], these frameworks are often specific to one phase of the medical education continuum. This specificity may limit generalizability to other types of health professional education as well as other stages of the medical education continuum. In addition, some frameworks, such as that developed by Akdemir and colleagues [6], focus on the purpose of the accreditation system and other macro-level characteristics, rather than detailed requirements and design characteristics. To date, there is no single, comprehensive framework for the operational design of medical education accreditation systems that outlines potential variations across systems or considerations for the alignment of design and development with local needs, contextual requirements, and stage of education.

This paper attempts to define a framework for the operational design of medical education accreditation that articulates design options as well as their contextual implications. Rather than espousing a “one size fits all” approach, our framework assumes that there is no single set of best practices in accreditation system development but, rather, an underlying set of *design decisions*. These decisions, when deliberated carefully, can ensure that an accreditation system is appropriately matched to the needs, resources, and contextual considerations of the local jurisdiction it serves as well as to the stage of education delivered by the program. Thus, our framework provides a means of designing systems that are a “best fit” for their local context.

## Main text

### A “fit for purpose” framework for accreditation system design

A “fit for purpose” approach aims to ensure that a system, policy, or program is designed and operationalized in a manner best suited to local needs and contexts. This approach is aligned with emerging models for education and international development that espouse decentralization. Specifically, rather than attempting to define a single approach or best practice, which may actually limit experimentation with and analysis of approaches tailored to a local, unique context [7], a focus on fitness for purpose or best fit can help ensure that different accreditation systems are “optimally adapted to [their] political, social and economic context” [8]. Given the complexity of medical education in today’s changing health care environments, a fit for purpose framework can also foster approaches that readily adapt to a jurisdiction’s changing situation over time.

During the group discussion at the World Summit, participants shared their experiences with accreditation processes in their local contexts, highlighting variations in practices and approaches for each accreditation element. Using the common framework outlined by Frank and colleagues [1], Table 1 summarizes the variations for each accreditation system element as identified by summit participants, supplemented by the authors’ knowledge and experience with health professions education accreditation systems internationally. For each accreditation system element, the discussion then considers implications for different models and variations, acknowledging that what might be optimal, or even practical, will depend on local factors such as the role or purpose of the accreditation system, the local regulatory context, and available resources. Ultimately, it is hoped that this paper will provide those who wish to develop a new or revised accreditation system for medical education within their jurisdiction with a flexible framework to guide their design decisions.

**Table 1:** A “fit for purpose” framework for medical education accreditation system design

Accreditation system element and definition	System sub-element	Variations across systems and contexts
**Mandate** The role and purpose of the accrediting body in reviewing the quality of educational programs, institutions, or systems.	Mandate	• Type of education or stage in the education continuum (undergraduate, postgraduate, or continuing professional development) • Role in the education system (mandatory vs. optional) and implications for learner certification, licensure, or maintenance of certification • Role of the accrediting body vis-à-vis government (legislated vs. non-legislated) • Focus of accreditation (QA vs. QI vs. mixed model) • Focus and scope of the accreditation system (national/local regulation vs. international comparison or benchmarking)
**Accreditation standards (criteria, requirements)** Measures or generally accepted benchmarks used in making decisions about the quality of a program, institution, or system	Standards taxonomies	• Types of criteria or benchmarks included in the standards (structures vs. processes vs. outcomes vs. mixed model) • Level of expectations (minimum standards vs. aspirational vs. mixed model) • Framework as a basis for standards content, including international taxonomies such as that used by WFME
	Process of standards development or renewal	• Content used for standards development and renewal (e.g., expert consensus, research evidence, government or regulatory directive, mixed model) • Cycle of standards evaluation and renewal (planned vs. unplanned) • Process of review (top down versus bottom up, who undertakes the review/evaluation, and who is involved [e.g., medical professionals, policy-makers, government, regulatory authorities, learners and/or graduates])
**Application for accreditation** The process of reviewing an initial request for accreditation by a program seeking to demonstrate compliance with established standards, and which results in a decision about whether to grant new (first-time) accreditation	Application process	• Timing (before learners enter the program vs. once they have entered or completed the program) • Application process (whether it differs from the regular accreditation process) and process elements (e.g., paper-based review, review by external assessors on site or by telephone) • Learner input (required vs. optional, or not included as part of the process) • Required follow-up after a successful application (a regular cycle of follow-up vs. shorter cycles for new programs)
	Requirements/ benchmarks for new accreditation	• Thresholds for a successful application (same threshold as established programs vs. a lower or higher threshold for new programs) • Focus of initial application (initial focus on structures/processes vs. focus on outcomes for more established programs) • Support to achieve new accreditation (education programs, coaching, or advisory services)
**Self-study (self-evaluation, self-assessment)** The internal process of reflection undertaken by a program, institution, or system to evaluate compliance with externally established standards	Self-study requirement	• Whether self-study is optional or mandatory
	Process of self-study	• Focus of self-study if required (on compliance with standards vs. descriptive narrative of program vs. emphasis on action plans to address identified areas for improvement) • Tools used for self-study (checklists vs. qualitative questionnaires vs. objective data [e.g., learner and/or graduate outcome data, survey data]) • Use of self-study if required (submission of the self-study to the accrediting body vs. use for the program’s own improvement only)
**External assessment of standards** The process of determining the level of compliance of a program, institution, or system with established accreditation standards, undertaken by individuals external to the program, institution, or system	Documentation	• Documentation available to inform the external assessment, such as results of program self-study, objective learner and graduate data (e.g., learner and/or graduate data, stakeholder feedback) or required documentation such as program policies • Timing of the documentation’s availability (before a site visit vs. at the site visit)
	External assessment process	• Basis of standards evaluation (validation of program’s self-study vs. objective review of program against standards) • Type of review (paper-based vs. review by tele- or video-conference vs. in-person site review) • Types of activities included in the review (review of documentation, site tours, meetings with key stakeholders, including learners and/or graduates)
**Accreditation report** The final report by external evaluators regarding the level of compliance of the program, institution, or system with established standards	Report content	• Tools to evaluate standards compliance (checklists, rating scales, narrative descriptions) • Type of report content (narrative/qualitative, vs. numerical/quantitative, vs. hybrid) • Process of report creation (automatically generated on the basis of standards evaluation vs. written report by external assessors or accrediting body staff)
**Accreditation decision** The final decision on accreditation status, and its associated follow-up, as determined by the accrediting body	Categories (types) of accreditation decisions (statuses)	• Types of accreditation status decisions, namely - binary (e.g., accredited/not accredited), - levels tied to degree of compliance attained (e.g., gold, silver, bronze) - levels tied to cycle length (e.g., 3 vs. 4 years), - levels tied to types of follow-up (e.g., regular accreditation review vs. focused review of follow-up report) • Whether there is a process to recognize and/or share innovations or program best practices
	Process of decision-making	• Criteria by which the decision is made (holistic judgment against overall criteria vs. established thresholds of compliance vs. evaluation of compliance with high-risk/minimum standards) • Who makes the decision (expert committee vs. accreditation body staff vs. computer) and how (group consensus vs. computer algorithm) • Information considered in making the decision (all available information vs. the accreditation only)
	Impact of and follow-up after the accreditation decision	• Whether the accreditation decision is considered final, versus an opportunity to improve the accreditation decision on the basis of new information or progress made • Impact of the accreditation decision and follow-up on learners within the program, e.g. on admissions, credentialing, certification, and maintenance of certification (see Mandate) • Process of appeal, including - what information is considered in the appeal, and - at what level an appeal is possible (individual standards vs. overall decision) • Transparency of the accreditation decision (public reporting/transparency vs. full confidentiality vs. hybrid [e.g., confidential details with published list of accredited programs])
**Accreditation cycle** The phases of an accreditation process dictating how often each program, institution, or system is re-evaluated for compliance with the standards, including the types of phases and activities in the process and any follow-up activities that must occur between external assessments	Types of accreditation cycles	• Length of cycle (e.g., 4, 6, or 8 years) • Whether the cycle is static for all programs or tied to the accreditation decision (longer for programs that demonstrate better compliance with standards) • Type of follow-up required (follow-up or special visit, regular accreditation process, progress report) • Types of activities throughout the cycle, ranging from a purely episodic cycle (once per cycle for full accreditation review) to more continuous cycles (e.g., those requiring regular submission of reports, activities, and/or data for ongoing monitoring)
**Site review model** The approach used by the accrediting body in determining the composition of its external site review team, as well as processes for recruiting, assigning, training, and assessing team members	Types of external reviewers (site reviewers, external assessors) and team composition	• Type of external site reviewer or external assessor used (volunteer/part-time vs. professional/full-time) • Expertise required by the site reviewer, e.g., peer review by physician or other health care professional, lay person with experience in accreditation, learners (students, residents, physicians in practice), mixed model • Size of the survey team (number of reviewers dedicated to each program or review) • Roles (whether there is a team lead or survey chair) • Role of the accrediting body’s staff in the site visit (observer only vs. full participant vs. process expert)
	Reviewer (assessor) training / professional development and assessment	• Types of training/professional development (workshops, mentorship programs, any formal training program and/or certification) • Whether surveyors are formally assessed and, if so, - types of assessment used (review by survey chair or team members, review by staff or a committee, and implications of negative assessments) - what is done with the assessment results (used to inform reviewers’ own development vs. used to inform future accreditation assignments)
**Accreditation system administration** The approaches used by the accrediting body to support the administration and operationalization of accreditation process; this component includes the business model, the technology used (if any), system review and improvement (including research and scholarship), and oversight and risk management	Technological infrastructure	• Little to no automation vs. systems with “electronic” paper vs. fully automated and digitized accreditation processes - automation may include internal scheduling/workflows, surveyor software, program software/portals, report generation software, technology to support ongoing data monitoring - different approaches to automation (“off-the-shelf” technological solutions vs. customized development)
	System improvement	• Approach to system improvement, from ad hoc to systematic, regular process of system review and improvement • Includes any focus on research and scholarship to improve the system of accreditation as well as that which is known about accreditation more generally
	Oversight and risk management	• Different approaches to oversight and risk management, including - committee governance - public accountability - legal risk management - regulation and established standards (accreditation of accrediting agencies, e.g. WFME, International Society for Quality in Healthcare)
	Business model	• Costs to accredited program or organization (free vs. annual fee or fee(s) associated with each accreditation activity) • Business model for the accrediting agency (funded by government or external funding vs. cost recovery vs. revenue generation, vs. mixed model)

### Elements of accreditation system design

Using the taxonomy as well the variations across different accreditation systems detailed in Table 1, we will now explore practical implications of the various elements of accreditation system design.

#### Accreditation system mandate

The mandate of a medical education accreditation system is determined by the type or stage of education it examines; its role in the education system as well as the role of the accrediting body itself; the focus of the accreditation system on QA or QI; and the scope of the accreditation system. These variables are interconnected. For example, the focus of the accreditation process on QA or QI (or a combination of both) may be driven by the particular type or phase of education that the accreditation system addresses, or by the role of the accreditation process in the system as a whole. Thus, accreditation systems for undergraduate medical education might be more structured or prescriptive than those for postgraduate programs; likewise, systems for postgraduate programs may be more structured or prescriptive than those for continuing professional development. Systems focused on training programs for more junior learners may require more restrictions, standardization, and protections for learners than those for more senior learners, particularly those already in unsupervised practice.

Further, when accreditation has implications for the certification and licensure of learners (e.g., when only graduates of accredited programs can be certified or licensed, or similarly, only accredited courses can be counted toward maintenance of certification), accreditation is often a mandatory or legislated requirement of the system. Conversely, accreditation is more likely to be optional in contexts where there is no implication for certification or licensure for graduates.

Finally, contexts in which accreditation is optional might be better aligned with a more aspirational approach focused on QI, whereas mandatory accreditation might be associated with a greater focus on minimum standards and QA. Likewise, systems that are focused on national or local regulation may be more likely to emphasize a QA approach, in contrast with systems that are international in nature, which may be better suited to a greater emphasis on QI or an aspirational philosophy.

### Accreditation standards

#### Standards taxonomies

Standards differ with respect to the types of criteria they include, as well as the level at which their expectations are set. These variations may be driven by the focus of the accreditation system (e.g., QA or QI) and the context of the education programs being accredited (e.g., under development or well established). For instance, requirements that focus on basic or minimum standards and processes may be best aligned with foundational accreditation systems, that is, those aimed at establishing an initial benchmark of quality, as well as with those focused on QA or minimum standards. In addition, standards that feature structure and process measures most prominently may be most essential when the standardization of training programs is the goal of the accreditation system or is important for the local context or the stage of education provided. For example, in systems where reciprocity of training between jurisdictions is highly important, structure and process measures in accreditation standards will help ensure standardization between programs.

Conversely, standards that emphasize outcomes, as well as those that are aspirational in nature, may be better suited to accreditation systems that permit flexibility with respect to program structures and processes, as well as those focused on QI. Increasingly, many accreditation systems are seeking a balance of structure, process, and outcome measures in their standards [9].

Internationally established standards frameworks may be best suited to accreditation systems with a focus on international benchmarking or comparison, but, depending on the heterogeneity of systems across jurisdictions and the specificity of the standards, they may lack local face validity or applicability. Conversely, locally developed standards can ensure better alignment with local contexts and requirements, particularly when substantial variation in those contexts necessitates a high degree of specificity in quality standards. On the other hand, context-specific standards may lack the benefit of comparability with other systems.

#### Process of standards development or renewal

Processes for the development and renewal of accreditation standards show substantial variation, particularly with regard to the content used for standards development, the process of review and renewal, and the cycle of standards evaluation and renewal. For example, many accreditation systems base their standards development or renewal on input from local experts as well as on consensus-based approaches, and this can help to ensure better face validity and acceptance on the part of the programs being evaluated against the standards. Conversely, standards development based on research evidence can have the benefit of identifying and incorporating innovations from other systems or sectors. Many accreditation systems rely on a mix of approaches, incorporating areas of innovation while still taking steps to ensure acceptance in the local context.

Cycles of standards evaluation and renewal may also depend on the resources available for standards development, and on what is practical in the local system or context. Appropriately, these cycles may also be driven by how fast the medical education system is changing in the local environment; for example, more frequent cycles of evaluation and renewal may be needed during periods of significant curricular change.

### Application for accreditation

#### Application process

An application process is not universal to all accreditation systems; however, it is typically seen in systems where some or all programs, providers, or institutions require a process for achieving new or first-time accreditation. An application process initiated by a program or institution often involves aspects or components typical of regular accreditation cycles, such as external assessment against standards, an accreditation report, and an accreditation decision; however, these processes are typically modified and separate from the accreditation system’s regular accreditation process for established programs or providers.

For new accreditation, where learner input is deemed essential before accreditation can be granted, the application process or initial accreditation may be deferred until after the first learners have entered or completed the program. It may also be advantageous to consider a two-part process: in the first stage, accreditation is granted contingently (“new” or “provisional” accreditation) on the basis of documentation and other information submitted before the program starts; this would be followed by a second application cycle after learners have completed all or part of the program.

In cases where the medical education system or program being accredited is new or not yet established, processes such as a pre-accreditation “readiness” assessment, an on-site or telephone-based review, or a shorter cycle of follow-up may provide better information about the program being accredited. The benefit of such processes may be especially clear in systems that contain widely differing programs, or where readiness for accreditation and compliance with the standards varies widely, such as in accreditation systems with an international scope.

Conversely, where the medical education system or program being accredited is well established or is less varied with respect to contexts or compliance with applicable standards, it may be possible to consider a paper-based review and a longer cycle of follow-up for the initial application.

#### Requirements or benchmarks for new accreditation

For new or first-time accreditation applications, the required or expected compliance with applicable standards must be established for accreditation to be granted. As shown in Table 1, accreditation systems differ in their pre-established thresholds or benchmarks; some require the same level of compliance as for pre-existing programs, while others allow for either a lower or higher threshold to achieve first-time accreditation. Some systems embed dedicated supports, such as coaching, into the process to help programs achieve first-time accreditation.

From a system design perspective, where the system or program seeking accreditation is new or not yet established, a lower threshold for a successful application may be justified. Likewise, a lower threshold for new applications as well as the availability of coaching and education services may be best aligned with accreditation systems focused on QI (vs. QA).

Conversely, where greater risks are associated with granting new accreditation, whether with respect to the learning environment or the implications of graduate certification for patient safety, an equal or higher threshold to achieve new accreditation may be warranted. This approach is more aligned with accreditation systems focused on QA or the establishment of a minimum standard of quality.

### Self-study (self-assessment, self-evaluation)

#### Self-study requirement

Accreditation systems differ with respect to their inclusion of a formal self-study component. The process of self-study is beneficial with respect to providing a framework for reflection, building a program’s knowledge of accreditation standards, and team building through meetings and discussions to self-evaluate compliance and consider opportunities for improvement.

Most accreditation processes for medical education include a self-study component, and this may be of particular importance in systems with a particular focus on QI (vs. QA). Whether the self-study process is required or optional may depend on factors, such as the maturity of the medical education system or the programs being accredited; local context; and whether a QI culture is well established. In the case of an optional self-study process, its use may be evaluated as a marker of the program’s own QI processes.

#### Self-study process and requirements

Self-study processes differ considerably among accreditation systems; considerations for self-study design include the focus of the self-study, the tools, and how the self-study is applied in the larger accreditation process.

The design of the self-study process may depend largely on the overall purpose of the accreditation system. Systems that emphasize or drive QI may benefit from the implementation of a self-study process; they may also wish to consider including mandatory standards that require programs to self-evaluate, monitor the outputs and outcomes of their activities, and develop action plans to address self-identified areas for improvement. Conversely, in systems where QA is the focus, self-study processes may still be beneficial but their use may be limited to helping programs identify and address deficiencies before the formal external evaluation. In still other systems, the self-study process may be part of the required documentation and a mandatory component of the accreditation process or review.

The results of a system or program’s self-study can provide additional information to the accrediting body in its evaluation of compliance with standards, and is often a required component of the accreditation process, particularly for earlier stages of the medical education continuum (i.e., undergraduate education) and in systems with a particular focus on QA. However, self-study information containing the program’s or institution’s own view of its compliance with standards can also bias the assessors’ evaluation. If other information is available to the assessors, e.g. policies, documentation, objective data about learners and/or graduates, etc., it may be a viable option to restrict certain content of the self-study to the program itself; this approach may encourage a more truthful or honest self-study, thereby promoting and encouraging better QI than would be the case if the program knows the self-study will be viewed by outside accreditors. This design consideration may be more applicable in systems that emphasize QI over QA, as well as those in later phases of the continuum.

Finally, the choice of tools for the self-study will depend on the study’s purpose and on how it will be used. If the self-study is intended for the program’s own QI, an accreditation system that offers variety or flexibility in the tools used may be advantageous. If, however, the self-study is intended to provide external assessors with information about the program to inform the evaluation of compliance against standards, the use of more objective data would likely be preferable.

### External assessment of standards

#### Documentation

The documentation used as part of an external assessment against standards varies from system to system according to the types of documentation available and the timing of their availability in the accreditation process. The choice of documentation to inform the external assessment should be driven largely by the requirements outlined in the pre-established accreditation standards. Optimally, multiple types of documentation should be available to allow for the triangulation of information and the accurate evaluation of standards compliance; however, the advantage of multiple types of documentation should be balanced with what is practical for the external assessors to incorporate into their review.

The timing of the availability of documentation may depend on what tools or infrastructure, e.g., an electronic platform, is available to transmit information in advance. Although providing documentation in advance can help with time management, it should also be considered whether this can be done easily and securely. In any case, the process for providing documentation for review by external assessors must ensure that learner information is kept confidential and is used only for its intended purpose.

#### External assessment process

The design of external assessment processes should be driven by the requirements outlined in the standards and by the scope of information needed to evaluate standards compliance; however, the processes can differ between systems with respect to how those standards are evaluated, as well as how the review is conducted, including which activities are included as part of the review.

Optimally, input from those most affected by the medical education system – the learners – should be included wherever possible. Methods for receiving input from learners will necessarily depend on the overall design of the external assessment process, but should if possible include confidential discussions with or other feedback from learners.

Although paper-based review has the advantage of not putting heavy demands on resources, it might not be possible to achieve an accurate evaluation of a program on this basis alone. External assessment should ideally include multiple types of data, evidence, and activities to allow for the triangulation of information and the accurate evaluation of standards compliance.

Conversely, where practically feasible, a physical on-site review can offer the most accurate evaluation of a program’s compliance with standards. A physical site review may be most important in situations where (1) the system of medical education and/or the program being accredited is new, or there has been a major change, such as curricular reform; (2) there is a need to review a facility or clinical learning environment; and (3) limited information is available to inform the evaluation of standards compliance. An on-site review allows for physical tours of clinical learning environments as well as of teaching environments. It also allows for discussions and interviews with program stakeholders to occur face to face, which can allow reviews to take non-verbal cues and body language into account. However, the practicality of the site review must be balanced with resource availability and other considerations, such as geographic distance or other factors which may limit travel and make telephone or video conferencing preferable. In systems where a site visit is difficult or impossible, the addition of other sources of information or evidence, e.g. a confidential report from learners or other stakeholders, regular survey tool administration and data collection, etc., may be helpful in rounding out the picture of standards compliance.

### Accreditation reports

#### Report content

Accreditation reports differs across systems according to the tools used to evaluate standards compliance, the type of content included in reporting, and how the reports are created. First and foremost, the choice of tools should be driven by the requirements outlined in the standards, as well as by the requirements and processes for reaching the final accreditation decision.

With respect to the report content, as well as the tools used to evaluate compliance, although objective, quantitative information has the advantage of being more easily reviewed and benchmarked, and of requiring little or no editing, it may also may miss the benefits associated with qualitative data, such as the richness of information captured in narrative descriptions. Purely quantitative reports may be best in contexts with fewer resources for report editing and where the emphasis is on QA and the attainment of minimum standards of quality. Reports based solely on quantitative data and information may also reduce legal or reputational risk to the accrediting organization and its assessors. In these cases, and where the technology is available to do so, accreditation reports can be generated automatically, with potential benefits for efficiency and accuracy.

Conversely, in systems that have resources for report editing, or where the focus of the accreditation system is on ongoing QI, and where there is limited legal or reputational risk to the accreditation organization (or where such risk can be mitigated), including qualitative information in the accreditation report can enhance the richness of the review and the feedback provided to institutions or programs. Ultimately, it may be best to aim for a hybrid of quantitative and qualitative reporting, balancing the advantages and disadvantages of each approach.

With respect to the process of report creation, this design decision will be driven largely by the type of content included in the report. As discussed above, reports that are based solely on quantitative information can be generated automatically from electronic checklists completed by the external assessors during the review. On the other hand, reports with a significant amount of qualitative information require writing and editing, whether by the external assessors themselves or by accreditation organization staff, during or after the visit.

### Accreditation decisions

#### Categories or types of accreditation decisions

Various types of accreditation decisions can be featured in an accreditation system. The types of accreditation status decisions are determined largely by other system design considerations, such as cycle length or the process of external assessment; however, it is important to differentiate between systems with binary categories of accreditation and those with multiple levels. Binary decisions may be appropriate in systems based on minimum standards of quality, which are either achieved or not achieved, and where the process is focused on QA rather than QI.

Conversely, in systems that focus on QI, designing categories of accreditation that include different levels to be achieved is an important way to encourage ongoing improvement and the attainment of aspirational standards. Levels of accreditation can also help assessors recognize those programs that have achieved the highest level of compliance with the standards. Similarly, processes to recognize and share innovations and best practices can encourage and create incentives and recognition for QI.

#### Process of accreditation decision-making

The process of rendering the final accreditation decision varies, as outlined in Table 1, according to the criteria upon which the decision is based, as well as by whether the decision is made by, for example, consensus, or by an automated process. The criteria used will depend on the requirements outlined in the standards as well as the types of accreditation decisions the process is intended to provide. Approaches based on established thresholds of compliance or the evaluation of high risk or minimum standards lend themselves well to systems with binary categories of accreditation status. These approaches can use a tool or checklist to evaluate compliance with each standard, rather than significant amounts of qualitative information; the process can also be facilitated by an electronic system or platform, potentially supported by a computer algorithm. This can help to standardize the process across programs as well as improve transparency.

Conversely, holistic judgments against overall criteria may be best aligned with systems for which the decision is complex or requires a significant volume of qualitative information. In these cases, arriving at a decision by the consensus of multiple experts can introduce checks and balances and reduce concerns about a lack of standardization; however, this approach can also lengthen the process and be resource-intensive.

Ultimately, it may be advantageous for many systems to consider a hybrid approach to decision-making, in which thresholds or weighted standards are used in conjunction with expert judgment to facilitate decision-making.

#### Impact and follow-up related to the accreditation decision

Typically, the process designed to reach an accreditation decision stipulates, implicitly if not explicitly, that the decision reached is final, barring any appeal. However, arguably, the opportunity to improve an accreditation decision on the basis of new information or progress made since the external assessment may promote an overall culture of QI and may be best aligned with more continuous (vs. episodic) accreditation cycles.

An appeal process is a necessary element of all accreditation systems to increase confidence in the process and the decisions rendered. The details of the appeal process may vary from system to system in light of legal frameworks in the jurisdiction and other contextual considerations.

The transparency of accreditation decisions, as well as detailed accreditation findings, is typically linked to the mandate of the accreditation system and its role within the medical education system. Mandatory systems as well as those that emphasize QA may tend toward greater transparency and public reporting of accreditation outcomes; conversely, systems that emphasize QI tend to emphasize the confidentiality of accreditation outcome information to promote a QI philosophy and culture. Hybrid approaches that share global accreditation outcomes without detailed findings may be an effective way to balance accountability, transparency, and even public protection within a QI approach.

### Accreditation cycle

#### Types of accreditation cycles

All accreditation processes require a defined cycle of review, distinguishing accreditation from one-time audits or other QA processes. However, as Table 1 highlights, accreditation cycles differ significantly between systems with respect to length, whether they are standardized for all accredited programs or variable according to the accreditation status, and types of activities used throughout the cycle, including any follow-up after the accreditation decision.

The type of accreditation cycle chosen should depend on contextual considerations regarding how much is known about the programs being accredited and how much uncertainty or risk there is within the medical education system and the programs being accredited. Where there is less risk or uncertainty, e.g., in a static educational curriculum versus a period of major curricular change, a longer accreditation cycle may be justified. Conversely, where there is more risk or uncertainty, a shorter accreditation cycle may be warranted, particularly in a purely episodic accreditation cycle with no required activities between regular accreditation visits. Likewise, more continuous systems with regular touchpoints or requirements throughout the accreditation cycle may increase confidence about program quality and allow the overall cycle to be lengthened.

In addition, in an accreditation system that emphasizes QI over QA, an approach that adjusts the length of the accreditation cycle length according to a program’s compliance with the standards may create incentives to strive for standards of excellence and/or markers of QI.

Finally, the typical length of an accreditation cycle may also depend on practical considerations, such as how many programs are accredited by the system and how many can feasibly be reviewed each year; the length of the education programs being accredited (shorter programs may justify shorter cycles of accreditation visits); and whether technology and other resources (such as staff) are available to support a more continuous approach to accreditation.

### The site-review model

#### Types of reviewers and team composition

Site review models differ according to the size and composition of the accreditation team. A peer review model wherein members of the profession serve as external assessors for programs other than their own (usually on a voluntary basis) may increase the face validity of the accreditation process. However, this advantage must be balanced against certain associated challenges, such as the need for a robust volunteer management program, as well as for rigorous site reviewer training and assessment to ensure consistency in the review process. Conversely, a site review model that uses professional, full-time surveyors can improve consistency without significant investments in volunteer management, training, and assessment. They method may, however, reduce face validity and confidence in the accreditation process overall.

In addition to the reviewers themselves, the accrediting body’s staff can play an important role, depending on the overall site review model used by the accrediting body. In the case of site review models based on volunteers and peer review, staff of the accrediting body can serve an important role by providing process expertise, thus helping to endure standardization. Conversely, site review models that rely on professional or staff surveyors may not benefit from or require additional staff support. In all cases, to mitigate potential conflicts of interest, staff who have a role in supporting the final accreditation decision-making process, including committee administration and the administration of appeal processes, should not be contributing participants in the accreditation review itself.

Finally, the size of the survey team depends largely on practical considerations such as how many program reviews are conducted each year and what type of external assessment is done.

#### Site reviewer training and assessment

The approach to training and assessing reviewers should be driven by the site review model as outlined above. Site review models based on volunteer peer review will require a more robust approach to training and assessment to ensure all reviewers meet an acceptable level of competence and to promote consistency in the accreditation process.

The approach to training and assessment may also depend on practical considerations, such as the availability of staff and resources. Training programs that rely on didactic sessions, potentially by means of electronic platforms such as learning management software or accreditation management systems, are effective for training large numbers of volunteers and can be easily duplicated. However, training that relies on the practical application of the accreditation process, although more resource and time intensive, will promote greater readiness and competence among new reviewers.

### Accreditation system administration

#### Technological infrastructure

The role of technology and other infrastructure in supporting the accreditation system is driven largely by practical considerations, such as the resources available to the accrediting body to invest in technology and automation, and whether automation can provide benefits to the accrediting body and those programs it accredits with respect to efficiencies, cost savings, and standardization. Some design options, such as more continuous accreditation processes supported by the regular submission of data or information, may be feasible only with the introduction of technological platforms.

Technological infrastructure may also be driven by strategic considerations, such as whether automation and an advanced technological platform can lend an advantage to accrediting bodies within a competitive environment. The use of such technology should also be guided by the “technological readiness” of the accrediting body, its stakeholders, and accredited programs. In systems where accreditation is new or the programs being accredited are untested, it may be preferable to introduce a simplified accreditation system that does not rely heavily on technology until such time as standards and accreditation processes are well embedded within the medical education system.

#### Accreditation system improvement

Approaches to the improvement of accreditation systems, as well as the approach to research and scholarship, vary from the ad hoc to the systematic. Cycles of system improvement and approaches to research depend largely on the resources available for review and improvement, and on what makes practical sense in the local system or context. As with standards themselves, the approach to overall system review and renewal should also be driven by how fast the medical education system is changing in the local environment; for example, more frequent cycles of evaluation and renewal may be needed during periods of significant curricular change.

#### Oversight and risk management

The accreditation system’s approach to oversight and risk management will depend largely on the context in which it works and on the need for such oversight and protection. However, a QA approach, in which accreditation decisions are more likely to be binary and to determine whether training can be counted for the purposes of high-stakes outcomes such as licensure, is more likely to be associated with systems that require additional protections in the form of risk management, governance, and oversight. A QA approach is also more likely to be expected or required to demonstrate accountability to the public and the profession.

#### Business model

The business model of an accreditation system is likely to depend on the role of accreditation in the local jurisdiction. For example, where accreditation is mandated or required by government, business models may be more likely to depend on government or other external funding, or to be funded through cost-recovery models.

Accreditation systems focused on national or local regulation may be more likely to have government involvement and funding, as opposed to those with an international mandate and scope, which may be more likely to have cost-recovery or revenue-generation business models.

### Limitations and future research

This “fit for purpose” framework to guide the development of new or renewed medical education accreditation systems is intended as a practical guide to exploring the implications and considerations of design decisions. We acknowledge that the framework and the considerations it engenders derive from expert opinion and experience rather than empirical evidence.

It would be useful to explore the application of this framework and its various design considerations across accreditation systems in a variety of jurisdictions, contexts, and types of health education, including different phases of the education continuum. This work could explore the design decisions that different systems make in light of practical considerations. This work would lead to a better understanding of variations across accreditation systems worldwide, as well as to further refinements to the framework itself.

## Conclusion

This “fit for purpose” framework for medical education accreditation system design builds on the common elements of medical education accreditation systems outlined by Frank and colleagues [1] and provides a principle-based framework, along with associated considerations and implications to take into account in developing and operationalizing any accreditation system for medical education programs.

The framework highlights that, rather than a single best practice, variation among accreditation systems is appropriate if it is based on and purposely designed to meet the needs of local contexts. In other words, form must follow function. This framework is intended to provide guidance to administrators, policy-makers, and educators regarding different approaches to medical education accreditation and their applicability and appropriateness in local contexts.

## Data Availability

Not applicable.

## Abbreviations

CQI: Continuous quality improvement
QA: Quality assurance
WFME: World Federation for Medical Education

## Footnotes

1 This discussion group, held at the 2013 World Summit on Outcomes-Based Accreditation in Calgary, Alberta, in conjunction with the International Conference on Residency Education, included representation from across the continuum of medical education (undergraduate, postgraduate, and continuous professional development) and from a number of international jurisdictions. These individuals represented a variety of roles in medical education and accreditation, including current and former program directors and deans, as well as accreditation surveyors and senior administrators. The list of participants is provided in Table 1 of Frank and colleagues [1].
